# The Latest Mania: Selling Bipolar Disorder

**DOI:** 10.1371/journal.pmed.0030185

**Published:** 2006-04-11

**Authors:** David Healy

## Abstract

Healy analyzes the surge in diagnoses of bipolar disorder and the evidence on the effectiveness of antipsychotic drugs and anticonvulsants in prophylaxis against the disorder.


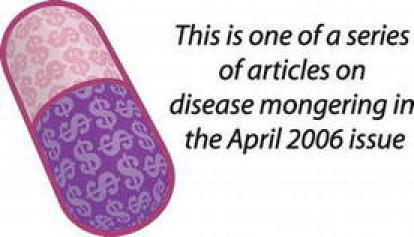


One of the most famous direct-to-consumer television adverts for a drug begins with a vibrant woman dancing late into the night. A background voice says, “Your doctor probably never sees you when you feel like this.” The advert cuts to a shrunken and glum figure, and the voiceover now says, “This is who your doctor usually sees.” Cutting again to the woman, in active shopping mode, clutching bags with the latest brand names, we hear: “That's why so many people with bipolar disorder are being treated for depression and not getting any better—because depression is only half the story.” We see the woman again depressed, looking at bills that have arrived in the post before switching to seeing her again energetically painting her apartment. “That fast- talking, energetic, quick tempered, overdoing it, up-all-night you,” says the voiceover, “probably never shows up at the doctor's office, right?”

No drugs are mentioned. But viewers are encouraged to log onto www.bipolarawareness.com, which takes them to a Web site called “Bipolar Help Center,” sponsored by Lilly Pharmaceuticals, the makers of olanzapine (Zyprexa). The Web site contains a “mood disorder questionnaire” ( http://www.bipolarhelpcenter.com/resources/mdq.jsp). In the television advert, we see our heroine logging onto www.bipolarawareness.com and finding this questionnaire. The voice encourages the viewer to follow her example: “Take the test you can take to your doctor, it can change your life….getting a correct diagnosis is the first step in treating bipolar disorder. Help your doctor to help you.”

This advert markets bipolar disorder. The advert can be read as a genuine attempt to alert people who may be suffering from one of the most debilitating and serious psychiatric diseases—manic-depressive illness. Alternatively, the advert can be read as an example of what has been termed disease mongering [ 1]. Whichever it is, it will reach beyond those suffering from a mood disorder to others who will as a consequence be more likely to see aspects of their personal experiences in a new way that will lead to medical consultations and in a way that will shape the outcome of those consultations. Adverts that encourage “mood watching” risk transforming variations from an emotional even keel into potential indicators of latent or actual bipolar disorder. This advert appeared in 2002 shortly after Lilly's antipsychotic olanzapine had received a license for treating mania. The company was also running trials aimed at establishing olanzapine as a “mood stabilizer,” one of which was recently published [ 2].

## Mood Stabilization

From the 1950s on, the depressions of manic-depressive illness have been treated with antidepressants and the manias with antipsychotics or lithium. Lithium was the only agent thought to be prophylactic against further episodes of manic-depressive illness [ 3]. But lithium was not originally referred to as a mood stabilizer. The term “mood stabilizer” had barely been heard of before 1995 when Abbott Laboratories got a license for using the anticonvulsant sodium valproate (Depakote) for treating acute mania [ 4].

**Figure 1:**
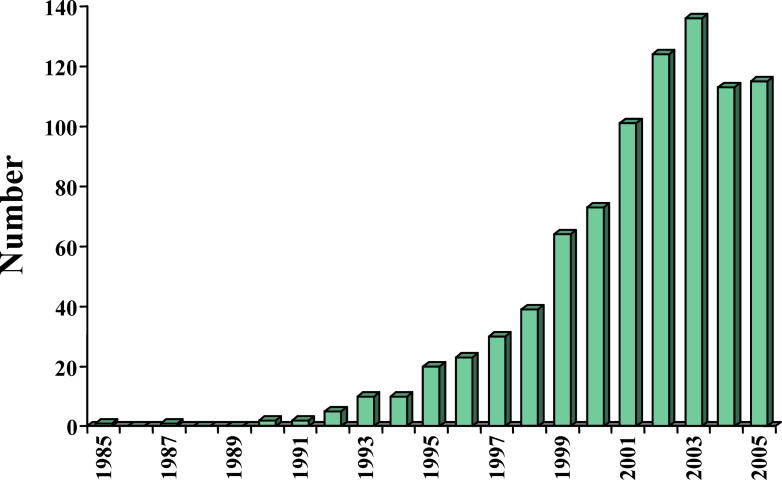
Articles Elicited by Medline Using the Mesh Term “Mood Stabilizer”

After 1995, there was a dramatic growth in the frequency with which the term “mood stabilizer” appeared in the title of scientific articles (see Figure 1). By 2001, more than a hundred article titles a year featured this term. Repeated reviews make it clear that the academic psychiatric community still has not come to a consensus on what the term “mood stabilizer” means [ 5–7]. But this lack of consensus did not get in the way of the message that patients with bipolar disorders needed to be detected and once detected needed mood stabilizers, and perhaps should only be given these drugs and not any other psychotropic drugs [ 8, 9].

The growth of awareness of mood stabilization was sensational.

The first group of drugs to colonize this new mood stabilizer niche was anticonvulsants. Anticonvulsants are beneficial in epilepsy and were until recently widely thought to be beneficial by quenching the increased risk of succeeding epileptic fits brought about by fits that have gone before. Robert Post in the 1980s suggested that anticonvulsants might stabilize moods by a comparable quenching of the kindling effect of an episode of mood disorders on the risk of further episodes [ 10]. It was this idea that provided a pharmacological rationale for treatment of bipolar disorders that was so attractive to pharmaceutical companies, and, in their hands, the growth of awareness of mood stabilization and of bipolar disorders was sensational.

Bipolar disorders entered the DSM *(Diagnostic and Statistical Manual of Mental Disorders)* in 1980. At the time, the criteria for bipolar I disorder (classic manic-depressive illness) involved an episode of hospitalization for mania. Since then, the community-based disorders bipolar II disorder, bipolar disorders NOS (not otherwise specified), and cyclothymia have emerged. With their emergence, estimates for the prevalence of bipolar disorders have risen from 0.1% of the population having bipolar I disorder (involving an episode of hospitalization for mania) [ 11] to 5% or more when the definition of bipolar disorders includes the aforementioned community disorders [ 12]. A range of academic institutions has also grown more interested in the condition.

There has always been a rationale to using antipsychotics in bipolar disorders, as they are effective in acute manic states [ 13, 14]. However, no companies making antipsychotics had previously sought a license for *prophylaxis* against bipolar disorders. Against a background of epidemiological studies indicating that the prevalence of bipolar disorders might be greater than previously thought [ 15, 16], and growing academic interest in the condition, Lilly, Janssen, and Astra-Zeneca, the makers of the antipsychotics olanzapine, risperidone, and quetiapine (Seroquel), respectively, marched in on the new territory to market these drugs for prophylaxis of bipolar disorder. This, in turn, greatly expanded the number of companies with an interest in making the “bipolar market.” There was, however, no consensus on a theoretical rationale that would lead the average clinician to think these three drugs might “quench” the propensity to further affective episodes, as opposed to simply assist in the management of acute manic states.

But the increased prevalence estimates were based on community surveys that had no clear disability criterion, while acute treatment trials of antipsychotics for mania, and prophylactic trials of lithium for manic-depressive illness, have for the most part been conducted on bipolar I disorder. This necessarily raises the prospect that increased efforts to detect and to treat people risks crossing the line where the benefits of treatment outweigh its risks.

Along with this expansion in prevalence estimates came new journals, *Bipolar Disorders* ( http://www.blackwellpublishing.com/journal.asp?ref=1398-5647) and the *Journal of Bipolar Disorders* (published by Lippincott, Williams, and Wilkins), a slew of bipolar societies, and annual conferences, many heavily funded by pharmaceutical companies. There is a growing amount of patient Web site and patient support materials that in the case of Zyprexa state that “bipolar disorder is often a lifelong illness needing lifelong treatment; symptoms come and go, but the illness stays; people feel better because the medication is working; almost everyone who stops taking the medication will get ill again and the more episodes you have, the more difficult they are to treat” [ 17]. Information available from Janssen (the makers of Risperdal) states “medicines are crucially important in the treatment of bipolar disorders. Studies over the past twenty years have shown beyond the shadow of doubt that people who receive the appropriate drugs are better off in the long term than those who receive no medicine” [ 18].

## What Lies Beneath

There is, however, much less evidence than many might think to support these claims for the prophylactic drug treatment of manic-depressive illness (bipolar I). And there is almost no evidence to support such claims in the case of whatever community disorders (bipolar II, bipolar NOS, cyclothymia) are now being pulled into the manic-depressive net by the lure of bipolar disorder.

With the possible exception of lithium for bipolar I disorder, there are no randomized controlled trials to show that patients with bipolar disorders in general who receive psychotropic drugs are better in the long term than those who receive no medicine [ 19]. This may stem in part from difficulties in conducting trials on psychotropic drugs that last more than a few weeks in conditions as complex as manic-depressive illness. One short-term, randomized, placebo-controlled trial (in which patients were only followed for up to 48 weeks) that some see as a basis for claiming that olanzapine may be prophylactic in bipolar disorder [ 2] has been regarded by others as indicating that this drug produces a withdrawal-induced decompensation when stopped [ 20]. Even in the case of lithium, there is some dispute over what has been demonstrated [ 19], with the best evidence stemming from large open studies in dedicated lithium services rather than from randomized trials [ 21].

This evidence of benefit for one agent (lithium) and possible benefit for one more (olanzapine) must be weighed against two harms associated with use of antipsychotics: (1) a consistent body of evidence indicates that regular treatment with antipsychotics in the longer run increases mortality [ 22–26]; and (2) there is evidence that in placebo-controlled trials of antipsychotics submitted in application for schizophrenia licenses there is a statistically significant excess of completed suicides on active treatment [ 27]. A range of problems associated with antipsychotics, from increased mortality to tardive dyskinesia, never show up in the short-term trials aimed at demonstrating treatment effects in psychiatry.

But aside from these hazards, there are also grounds to question whether the treatment effects that some think have been demonstrated in bipolar disorder trials translate into therapeutic efficacy. If use of these agents based on demonstrated effects leads on to efficacy, admissions for bipolar disorder might be expected to fall, but the evidence for this is difficult to find. In North Wales before the advent of modern pharmacotherapy, patients with bipolar I disorder had on average four admissions every ten years. In contrast, against a background of a constant incidence of bipolar I disorder, and dramatic improvements in service provision, bipolar I patients show a 4-fold increase in the prevalence of admissions despite being treated with the very latest psychotropic medications [ 11]. This is not ordinarily what happens when treatments “work,” but quite often is what happens when treatments have effects.

The selling of bipolar disorder stresses that the disorder takes a fearsome toll of suicides. And indeed the controversy surrounding the provocation of suicide by antidepressants has been recast by some as a consequence of mistaken diagnosis. If the treating physician had only realized the patient was bipolar, they would not have mistakenly prescribed an antidepressant. Because of the suicide risk traditionally linked to patients with bipolar disorders who needed hospitalisation, most psychiatrists would find it difficult to leave any person with a case of bipolar disorder unmedicated. Yet, the best available evidence shows that unmedicated patients with bipolar disorder do *not* have a higher risk of suicide.

**Figure 2:**
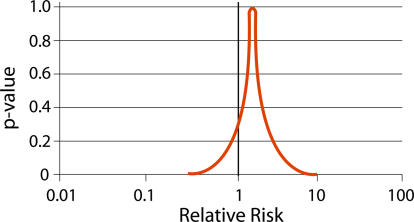
Author's Graph of *p*-Value Function Based on Data in [ 30] (Illustration: Sapna Khandwala)

Storosum and colleagues analyzed all placebo-controlled, double-blind, randomized trials of mood stabilizers for the prevention of manic/depressive episode that were part of a registration dossier submitted to the regulatory authority of the Netherlands, the Medicines Evaluation Board, between 1997 and 2003 [ 28]. They found four such prophylaxis trials. They compared suicide risk in patients on placebo compared with patients on active medication. Two suicides (493/100,000 person- years of exposure) and eight suicide attempts (1,969/100,000 person-years of exposure) occurred in the group given an active drug (943 patients), but no suicides and two suicide attempts (1,467/100,000 person-years of exposure) occurred in the placebo group (418 patients). Based on these absolute numbers from these four trials, I have calculated (see Figure S1 showing calculation, and see Figure 2) that active agents are most likely to be associated with a 2.22 times greater risk of suicidal acts than placebo (95% CI 0.5, 10.00).

## The Bipolar Future

Until recently the general clinical wisdom was that it was very rare for manic-depressive illness to have an onset in the preteen years. But there is now a surge of diagnoses of bipolar disorder in American children [ 29, 30], even though these children do not meet the traditional criteria for bipolar I disorder (from the *Diagnostic and Statistical Manual of Mental Disorders*) [ 31]. The mania for pediatric bipolar disorder hit the front cover of the American edition of *Time* in August 2002, which featured nine-year-old Ian Palmer and a cover title *Young and Bipolar*, with a strapline, *why are so many kids being diagnosed with the disorder, once known as manic-depression?*

A recent book, *The Bipolar Child* [ 32], brings out the extent of the current mania. Published in 2000, this book sold 70,000 hardback copies in six months in the US. As the *Star Telegram* reported in July 2000 [ 33], *The Bipolar Child* made all the difference to Heather Norris, whose mother, after reading it, challenged her physician to correct Heather's diagnosis from ADHD, treatment of which had made her daughter worse, to the correct diagnosis of bipolar disorder. As a result, Heather, at the age of two, became the youngest child in Tarrant County, Texas, to have a diagnosis of bipolar disorder. The *Star Telegram* article noted that “along with the insurance woes, lack of treatment options and weak support systems that plague most families with mentally ill children, parents of the very young face additional challenges. Finding the proper diagnosis for treatment is a nightmare because of scant research into childhood mental illness and the drugs that combat them.”

If we consider adults alone for a moment, there is already the potential for creating an “epidemic” of bipolar disorder, because people are being diagnosed with the condition based on operational criteria that depend upon subjective judgements (rather than an objective criterion of disability, such as hospitalization or being off work for a month). The potential is compounded in the pediatric domain by the fact that the diagnosis is based on caregiver reports with little scope in most clinical practice for critical scrutiny of the social forces that may lead to these reports. Experts that appear willing to go so far as to accept the possibility that the first signs of bipolar disorder may be patterns of overactivity in utero [ 32] can only further compound these problems. If the resulting diagnoses were provisional, aimed at researching the natural history of childhood irritability, rather than reaching diagnoses that lead on to pharmacotherapy, there might be little problem. However, drugs such as Zyprexa and Risperdal are now being used for preschoolers in America with little questioning of this development [ 31].

Far from research bringing a skeptical note to bear on clinical enthusiasm, it appears to be adding fuel to the fire. What might once have been thought of as sober institutions, such as Massachusetts General Hospital, have run trials of Risperdal and Zyprexa on children with a mean age of four years old [ 34, 35]. Massachusetts General Hospital in fact recruited trial participants by running its own television adverts featuring clinicians and parents alerting parents to the fact that difficult and aggressive behavior in children aged four and up might stem from bipolar disorder. This does more than recruit patients with a clear disorder; it suggests that everyday behavioral difficulties may be better seen in terms of a disorder. Given that bipolar disorder in children is all but unrecognised outside the US, it seems likely that a significant proportion of these children will not meet conventional DSM criteria for bipolar I disorder. And given that it is all but impossible for a short-term trial of sedative agents in pediatric states characterized by overactivity *not* to show some rating scale changes that can be regarded as beneficial, the outcomes of this research are likely to appear to validate the diagnosis and increase the pressure for treatment.

Several years after Heather Norris was diagnosed with bipolar disorder, the rationale for mood stabilization was greatly weakened by the results of the largest-ever randomized trial of immediate versus deferred anticonvulsant therapy for people who had experienced a single seizure [ 36]. The trial found that although immediate antiepileptic drug treatment reduces the occurrence of seizures in the next 1–2 years, such treatment does not affect long-term remission in individuals with single or infrequent seizures. The use of psychotropic medication for bipolar disorders was based on an analogy with epilepsy, rather than on demonstrations of proven clinical benefits over the long term or on the basis of a correction of a known pathophysiology. The absence of a solid theoretical or empirical basis for using psychotropic medication as “mood stabilizers” raises questions as to what lies in store for the Heather Norris's and others of this world exposed to these complex psychotropic agents from such a young age.

## Supporting Information

Figure S1 Episheet Showing Author's Relative Risk Calculation, Based on Data in [ 30]
(792 KB XLS).
