# Correction: The Latest Mania: Selling Bipolar Disorder

**DOI:** 10.1371/journal.pmed.0030236

**Published:** 2006-05-30

**Authors:** David Healy

In *PLoS Medicine*, volume 2, issue 4: DOI: 10.1371/journal.pmed.0030185

The correct title for Figure 2 should be “Author's Graph of *p*-Value Function Based on Data in [28].” The correct title for Figure S1 should be “Episheet Showing Author's Relative Risk Calculation, Based on Data in [28].”

